# Epidemiology of gaming disorder and its effect on anxiety and insomnia in Chinese ethnic minority adolescents

**DOI:** 10.1186/s12888-022-03894-3

**Published:** 2022-04-12

**Authors:** Qiaoyue Wei, Shengjie Zhang, Yuli Pan, Hong Hu, Fenglan Chen, Wenwen Yin, Qinghong Lin, Shuibo Pan, Chingyuan Tham, Junduan Wu

**Affiliations:** 1 Department of Psychology, School of Public Health, Guangxi Medical University, 22 Shuangyong Road, Nanning, 530021 China; 2 Department of Graduate Management, Guangxi Medical University Cancer Hospital, 71 Hedi Road, Nanning, 530021 China; 3 Department of Guangxi Zhuang Autonomous Region Center for Disease Control and Prevention, 18 Jinzhou Road, Nanning, 530028 China; 4 Department of Guangxi Medical University Wuming Hospital, 26 Yongning Road, 530199 Nanning, China

**Keywords:** Gaming disorder, Effect, Anxiety, Insomnia, Adolescents

## Abstract

**Background:**

The growing popularity and frequency of online game use have resulted in a large number of studies reporting various mental problems associated with game abuse in adolescents. In this article, we examined the prevalence of gaming disorder (GD) and explored the associations of GD with anxiety and insomnia symptoms in minority youth in China.

**Methods:**

A total of 1494 students completed the Problematic Online Gaming Questionnaire Short-Form (POGQ-SF), the Generalized Anxiety Disorder 7-item questionnaire (GAD-7), and Athens Insomnia Scale (AIS). Chi-square and binary logistic regression analyses were used to explore the associations between gaming disorder and anxiety/insomnia.

**Results:**

A total of 356 (23.83%) respondents reported that they had gaming disorder. Chi-square analysis showed that gender, grade, marital status of parents and exercise situation were significantly associated with GD. Binary logistic regression analysis showed that those who had GD were at significantly higher risk for anxiety and insomnia than those without GD.

**Conclusion:**

We found a high incidence of GD and a positive association among anxiety, insomnia and GD. Thus, special attention should be paid to those who have suffered from GD. It is worth addressing the adverse effects of GD on anxiety and insomnia.

## Background

For adolescents, online gaming has become a centrally important sociocultural practice [1]. Despite the positive cognitive functions of gaming [2], most scholars agree that it is potentially harmful to pathologically use video games, causing negative consequences for adolescents [3]. Recently, the World Health Organization (WHO) included gaming disorder in their International Classification of Diseases (ICD-11) [4]. Gaming disorder (GD) is defined as a pattern of gaming behavior (“digital gaming” or “video gaming”) characterized by impaired control over gaming, increasing the priority given to gaming over other activities to the extent that gaming takes precedence over other interests and daily activities; and continuation or escalation of gaming despite the occurrence of negative consequences [5]. The prevalence of GD in adolescents was reported to be between 0.6% and 19.9% in a recent meta-analysis [6]. Studies have reported that young age and male sex are risk factors for GD [7]. Furthermore, adolescents exposed to unstable parental relations and parental absence, which indicate low family function or left behind children, are more vulnerable to GD [8–11]. Similarly, status as an only child is more likely to result in GD than other statuses [12, 13]. A recent meta-analysis reported that GD is positively correlated with many physical health–related effects, such as somatization, hand and wrist pain, decreased levels of physical activity, cardiovascular stress reaction, and sleep problems [14]. These harmful influences will interfere with daily life activities [15]. Additionally, GD behaviors can result in a series of negative psychosocial repercussions, including poor cognitive function (e.g., memory, attention), impaired socializing, depression, anxiety, and other addictions [16–20]. Furthermore, chronic physical illness has been found to be associated with an increased risk of mental health problems [21], and it is particularly significant to study the psychological problems related to GD. Therefore, the past few decades have seen increased research interest in GD, and a number of research teams have made substantial efforts to define the prevalence of GD and related mental problems [22]. However, few prior studies have examined the effects on anxiety and insomnia among Chinese ethnic minority adolescents. Therefore, it is crucial to evaluate the influence on ethnic minority adolescents.

As one of the most common mental disorders during adolescence, anxiety’s prevalence is high among this age group [21]. Anxiety can be amplified by reductions in real-world confidence, creating negative emotional states that impede real-life social interactions and task completion [23]. One study found that the degree of addictive video game use was related to anxiety disorders [24]. Furthermore, potential factors of anxiety have been identified as well, such as age, gender and parental absence [25–27].

Insomnia problems have become a challenge for adolescents worldwide. Previous studies have found that insomnia starts to emerge during adolescence, with the prevalence ranging from 19.3% to 40% in the young population [28, 29]. Adolescent insomnia tends to be chronic and frequently coexists with or precedes the onset of psychiatric illnesses [30]. There was a considerable systematic review in 2014 showing that insomnia could lead to a number of negative consequences, including daytime impairments, poor academic performance, pain, cardiovascular and cardiometabolic impairments, and mental/emotional dysfunction [31]. In addition, GD, age (≥ 16 years old), sex (female) and parental absence have also been considered risk factors for insomnia [32–34]. In a word, studies have shown that GD is related to anxiety and insomnia. Furthermore, there are some likely shared biological mechanisms. The blue light emitted from a display such as a laptop or smartphone could play a role in sleep disturbances and anxiety levels by altering melatonin [35–37]. In addition, various studies have found significantly increased sympathetic activity in participants with internet addiction [38, 39]. The both pathways can also affect sleep quality and anxiety disorder [40, 41].

A number of studies have investigated the prevalence of excessive gaming and explored its relationship with mental and physical issues among adolescents globally, but similar research in Chinese ethnic minorities has not been perfromed. Previous studies have shown that girls in ethnic minority areas suffer significantly higher rates of physical, mental and neglect than those in Han areas in China, negatively affecting their mental health at different levels [42]. Moreover, the majority of ethnic minority Zhuang adolescents live in southern China, where they have their heritage language, cultural beliefs, and educational values. In addition, before starting high school, most of the Zhuang adolescents live in a distant rural location far from the school grounds [43, 44]. All of these challenges might place Zhuang children in a very disadvantaged situation regarding mental health [45], such as excessive gaming to escape the barrier of language communication. Therefore, it is important to explore the relationship between GD and anxiety/insomnia in ethnic minority adolescents. This study primarily aimed to investigate the prevalence of GD in ethnic minorities. The second purpose was to describe the differences in gender, grade, marital status of parents, exercise situation, only child status, place of residence, parental absence situation and type of school in these GD groups. The third purpose was to analyze the relationships of GD with anxiety and insomnia and to identify related risk factors.

## Methods

### Participants

A cross-sectional study was conducted in September 2019 at three schools (two junior highs and a senior high school) in Guangxi, China, and was enrolled via stratified cluster sampling. First, we randomly selected 3 campuses in Guangxi. Second, we randomly selected 10 classes from the class roster. Third, we invited all students to complete a questionnaire in a classroom during class time and collected it in class. All methods were thoroughly explained to each participant, and all of the participants provided written informed consent to participate in the study. Excluding those who refused to participate in the study, a total of 1604 students were recruited for our study. Among the respondents, 110 were excluded due to incomplete data. Finally, 1494 students’ questionnaires were qualified, and the actual response rate was 93.14% (1494/1604). The inclusion criteria were as follows: 1) aged 12–18 years old; 2) Zhuang nationality; and 3) no history of mental disorders or severe physical illness. The sample included 764 (48.8%) junior high school students and 730 (51.1%) senior high school students. Among them, 712 (47.6%) were male, and 782 (52.3%) were female. The average age of the respondents was 14.9 ± 1.5 years old. This study was conducted following the Declaration of Helsinki and was approved by the Institutional Ethical Committee of Guangxi Medical University (approval number: 20160302–13).

### Measurements

#### Participants’ general information

A questionnaire was used to collect basic information regarding age, grade (senior high school/junior high school), gender (male/female), marital status of parents (stable/unstable), place of residence (urban/rural), status as an only child (yes/no), type of school (urban school/suburban school/rural school), parental absence (‘Did either of your parents ever leave home before you were 18 years old?’ yes/no), and exercise situation (‘Did you exercise in the past week’ yes/no).

#### Problematic Online Gaming Questionnaire Short-Form (POGQ-SF)

The scale consists of 12 items. Each entry is scored five points: "1" means "never", "2" means "very little", "3" means "occasionally", "4" means "often", and "5" means "always". A score of 32 points is an optimal cutoff to classify disordered gaming as problematic gaming [46]. The application of the POGQ-SF has good reliability and validity in China [47] and foreign countries [48]. The current sample for the POGQ-SF was calculated using Cronbach’s alpha with a value of α = 0.869.

#### Generalized Anxiety Disorder (GAD-7)

The scale consists of 7 items, which are mainly used as screening tools for anxiety disorders. It can be used to evaluate the severity of anxiety symptoms: 0–4 points for no clinical significance of anxiety, 5–9 points for mild, 10–14 for moderate, and more than 15 points for severe [49]. The current sample for the POGQ-SF was calculated using Cronbach’s alpha with a value of α = 0.882.

#### Athens Insomnia Scale (AIS)

The AIS was developed by Soldatos and colleagues to assess the severity of insomnia based on the ICD-10 diagnostic criteria. It is a self-report questionnaire consisting of 8 items; the first 5 items assess difficulty with sleep induction, awakening during the night, early morning awakening, total sleep time, and overall quality of sleep, while the last 3 items pertain to the sense of well-being, overall functioning and sleepiness during the day. The usual time frame for responding is the previous month. Each item of AIS can be rated 0–3, with 0 corresponding to no problem at all and 3 to a very serious problem. The Chinese versions of AIS is reliable and valid instrument of insomnia in adolescents, a cut-off score of 7 points (sensitivity/specificity: 0.78/0.74) is an optimal cut-off to classify insomnia (Cronbach’s α = 0.81) [50]. In this study, the Cronbach’s α = 0.794.

#### Statistical analysis

The data were analyzed with SPSS Statistics software, version 23 (IBM, Armonk, NY, USA). Chi-square and binary logistic regression analyses were used to explore the associations between gaming disorder and anxiety/insomnia. *P* values less than 0.05 were considered statistically significant.

## Results

### The prevalence of gaming disorder and general demographic data

Of the 1494 participants, 1138 (76.17%) reported that they had no gaming disorder, and 356 (23.83%) reported that they had gaming disorder. Those who had gaming disorder had the highest rate of male gender (35.67%), followed by those whose parental relations were unstable (32.60%), who did not exercise (31.64%) and those who studied in rural schools (28.16%). Table 1 summarizes these results.

**Table 1:** Demographic characteristics of gaming disorder

Variables	Total	gaming disorder		χ^2^
		No	Yes	
Gender				105.158***
Male	712	458 (64.33)	254 (35.67)	
Female	782	680 (86.96)	102 (13.04)	
Grade				5.299*
Senior high school	730	575 (78.77)	155 (21.23)	
Junior high school	764	563 (73.69)	201 (26.31)	
Marital status of parents				
Stable	1313	1016 (77.38)	297 (22.62)	8.723**
Unstable	181	122 (67.40)	59 (32.60)	
Only child				3.155
Yes	319	231 (72.41)	88 (27.59)	
No	1175	907 (77.19)	268 (22.81)	
Place of residence				0.000279
Rural	1122	854 (76.11)	267 (23.80)	
Urban	372	284 (76.34)	89 (23.92)	
Parental absence				3.675
Yes	845	628 (74.32)	217 (25.68)	
No	649	510 (78.58)	139 (21.42)	
Exercise situation				11.320**
Yes	1219	950 (77.93)	269 (22.07)	
No	275	188 (68.36)	87 (31.64)	
Type of school				6.285*
Urban school	730	576 (78.97)	154 (21.10)	
Suburban school	519	387 (74.57)	132 (25.43)	
Rural school	245	176 (71.84)	69 (28.16)	

^*^p < 0.05

^**^p < 0.01

^***^p < 0.001

### Single-factor analysis of anxiety/insomnia

In terms of anxiety levels, 762 (51.0%), 499 (33.4%), 147 (9.8%), and 86 (5.7%) respondents displayed no, mild, moderate, and severe levels of anxiety, respectively. We used the chi-square test to determine differences in anxiety levels among variables. Statistical significance was found among anxiety levels, gaming disorder, gender, parental absence and type of school (Table 2).

**Table 2:** Differences in anxiety/insomnia status according to variables

Variable	Total	Anxiety				χ^2^	Insomnia		χ^2^
		No	Mild	Moderate	Severe		No	Yes	
Gaming disorder						52.890***			77.484***
No	1138	632 (55.5)	363 (31.8)	95 (8.3)	48 (4.2)		594 (52.2)	544 (47.8)	
Yes	356	130 (36.5)	136 (38.2)	52 (14.6)	38 (10.6)		91 (25.6)	265 (74.4)	
Gender						44.284***			8.201**
Female	782	339 (43.3)	287 (36.7)	97 (12.4)	59 (7.5)		331 (42.3)	451 (57.7)	
Male	712	423 (59.4)	212 (29.7)	50 (7.0)	27 (3.7)		354 (49.7)	358 (50.3)	
Grade						6.157			0.117
Junior high school	764	407 (53.2)	233 (30.4)	80 (10.4)	44 (5.7)		347 (45.4)	417 (54.6)	
Senior high school	730	355 (48.6)	266 (36.4)	67 (9.1)	42 (5.7)		338 (46.3)	392 (53.7)	
Marital status of parents						6.229			10.117**
Stable	1313	683 (52.0)	430 (32.7)	123 (9.3)	77 (5.8)		622 (47.4)	691 (52.6)	
Unstable	181	79 (43.6)	69 (38.1)	24 (13.2)	9 (4.9)		63 (34.8)	118 (65.2)	
Exercise situation						6.876			6.540*
Yes	1219	630 (51.6)	413 (33.8)	110 (9.0)	66 (5.4)		578 (47.4)	641 (52.6)	
No	275	132 (48.0)	86 (31.2)	37 (13.4)	20 (7.2)		107 (38.9)	168 (61.1)	
Only child						6.551			1.096
Yes	319	176 (55.1)	89 (27.8)	31 (9.7)	23 (7.2)		138 (43.3)	181 (56.7)	
No	1175	586 (49.8)	410 (34.8)	116 (9.8)	63 (5.3)		547 (46.6)	628 (53.4)	
Place of residence						4.193			0.183
Rural	1122	588 (52.4)	361 (32.1)	111 (9.8)	62 (5.5)		518 (46.2)	604 (53.8)	
Urban	372	174 (46.7)	138 (37.0)	36 (9.6)	24 (6.4)		167 (44.9)	205 (55.1)	
Parental absence						8.374*			1.434
Yes	845	411 (48.6)	284 (33.6)	97 (11.4)	53 (6.2)		376 (44.5)	469 (55.5)	
No	649	351 (54.0)	215 (33.1)	50 (7.7)	33 (5.0)		309 (47.6)	340 (52.4)	
Type of school						25.037***			1.596
Urban school	730	355 (48.6)	266 (36.4)	67 (9.2)	42 (5.8)		338 (46.3)	392 (53.6)	
Suburban school	519	303 (58.4)	146 (28.1)	46 (8.9)	24 (4.6)		228 (43.9)	291 (56.1)	
Rural school	245	104 (42.4)	87 (35.5)	34 (13.9)	20 (8.2)		119 (48.6)	126 (51.4)	

^*^p < 0.05

^**^p < 0.01

^***^p < 0.001

In addition, the chi-square test was also used to determine differences in insomnia levels among variables. In terms of insomnia levels, 685 (45.85%) and 809 (54.15%) respondents displayed no insomnia and some level of insomnia, respectively. Statistical significance was found among insomnia levels, gaming disorder, gender, marital status of parents and exercise situation (Table 2).

### Multivariate logistic regression analysis of anxiety/insomnia

For anxiety, the chi-square test results of statistically significant variables were included in the multivariate logistic regression analysis. The dependent variable was anxiety level, whereas the independent variables were gaming disorder, gender, parental absence and type of school. The normal level of anxiety was used as the reference level. For all anxiety groups, gaming disorder and gender were positively related (OR: 1.689–3.849, all *p* < 0.001). In addition, parental absence was positively related to moderate anxiety (OR = 1.657, *p* < 0.05). Studying in urban schools (OR = 0.412–0.576, all *p* < 0.05) was negatively related to all severe anxiety groups relative to those who studied in rural areas (Table 3).

**Table 3:** Adjusted odds ratios (aORs) for anxiety/insomnia associated with relevant variables and gaming disorder

Variable	Anxiety			Insomnia
	Mild	Moderate	Severe	Yes
	aOR (95% CI)	aOR (95% CI)	aOR (95% CI)	aOR (95% CI)
Gaming disorder				
No	1	1	1	1
Yes	1.821 (1.386,2.393)***	2.661 (1.807,3.919)***	3.849 (2.416,6.131)***	3.180 (2.439,4.145)***
Gender				
Male	1	1	1	1
Female	1.689 (1.345,2.122)***	2.421 (1.672,3.504)***	2.727 (1,692,4.395)***	1.347 (1.098,1.653)**
Marital status of parents				
Stable	—	—	—	1
Unstable	—	—	—	1.686 (1.219,2.332)**
Exercise situation				
Yes	—	—	—	1
No	—	—	—	1.416 (1.084,1.849)*
Parental absence				
No	1	1	1	—
Yes	1.128 (0.899,1.416)	1.657 (1.145,2.398)*	1.372 (0.868,2.167)	—
Type of school				
Rural school	1	1	1	
Suburban school	0.896 (0.646,1.241)	0.577 (0.362,1.121)	0.615 (0.346,1.094)	—
Urban school	0.576 (0.407,0.815)**	0.464 (0.283,0.763)*	0.412 (0.219,0.776)**	—

^*^p < 0.05

^**^p < 0.01

^***^p < 0.001

For insomnia, the chi-square test results of statistically significant variables were included in multivariate logistic regression analysis. The dependent variable was insomnia level, whereas the independent variables were gaming disorder, gender, marital status of parents and exercise situation. The normal level of insomnia was used as the reference level. For insomnia, gaming disorder, gender, marital status of parents and exercise situation were positively related (OR: 1.374–3.180, all *p* < 0.05). Those who had gaming disorder (OR = 3.180, *p* < 0.001) were at significantly higher risk for insomnia than those who did not have gaming disorder (Table 3).

## Discussion

### Prevalence and demographic distribution of gaming disorder

Our study found that approximately 24% of Chinese adolescents have gaming disorder. This result seems to be significantly higher than a previous study reported in South Korea, which showed that the rate of internet addiction in the population was approximately 20.3% (using IAT) [51]. This finding also means that the prevalence reveals an increasing trend year by year [52]. One of the possible explanations for the assessment tool is different. Exploration is needed when drawing comparisons among different countries and periods of survey due to the complexity of GD and linguistic differences, in addition to the variations in assessment tools and criteria. Although our study areas are the same place, the prevalence varied according to demographics: there was a very high prevalence of gaming disorder among male respondents, consistent with a past study suggesting that males are at higher risk than females [10, 53]. This finding suggests that men might be more vulnerable to addiction in general [51]. However, the situation seems to have changed [54]. For instance, in 2020, Arpaci revealed that female undergraduate students had significantly higher GD scores than male students [55], and Chung et al. reported that females were at a significantly higher risk for smartphone addiction [56]. Tang et al. reported that the prevalence of social networking addiction in males and females was 27.8% and 37.3%, respectively [57]. There might be an important reason for this phenomenon: female students mainly use the internet for social interaction, while male students prefer to use the internet for online games [58]. In addition, those who reported that parental relations were unstable also had a high incidence of GD. In an unstable family environment, which means there is limited time to build a relationship with the children, adolescents might resort to accessing the internet to relieve psychological insecurity [59]. Furthermore, adolescents who do not exercise are more vulnerable to internet addiction (IA). This fact is not surprising given that this group lacks face-to face communication and likely stays indoors, causing mobile phones to become a more important part of their lives, leading to the occurrence of gaming disorder. Moreover, the prevalence of GD in different types of schools (21.10%, 25.43%, 28.16%) within China differs because of imbalances in the levels of development. The government should pay more attention to underdeveloped regions and rural schools [60].

### Association between gaming disorder and anxiety

Our study found that GD was a very strong risk factor for anxiety (whether mild, moderate or severe); this result is similar to previous research showing that anxiety symptoms were positively related to the severity of GD [61]. Youths with IA also reported more anxiety symptoms [62]. Maladaptive and excessive internet use can result in or further amplify anxiety symptoms [63]. It is not surprising that higher rates of anxiety are found among GD users as a result of a perceived and perhaps overwhelming obligation to remain constantly connected to various online games through their phones [64]. Because the internet and games are ever present, it might be difficult for some addiction users to disconnect. Additionally, the reason for this finding could be that overuse of the internet can be seen as a rewarding behavior, and it can be employed as an insufficient coping strategy against anxiety by learning mechanisms [65]. People with GD might consequently isolate themselves and neglect to engage in behavioral activity that is important to psychological health [66]. As a result of such social isolation and decreased behavioral activity, people with GD might suffer from anxiety symptoms as a consequence. Our finding that being female was a risk factor for anxiety was similar to a study that found that female students suffered from significantly higher levels of anxiety than male students [67]. The anxiety score of any given person is the product of both biological and psychological factors and their interaction [68]. It is not only that female respondents might be more likely to report their symptoms but also that the cyclical fluctuations of estrogens enhance the response to stress, conferring susceptibility to depression and anxiety. Another finding was that parental absence was also a risk factor for moderate anxiety. In addition, we believe that parental absence could lead to GD because the absence of parents leads to adolescents lacking control over internet use, resulting in internet addiction. As Yen reported, all gaming disorder users’ behaviors and anxiety are influenced by family interactions, and GD increases the risk of anxiety [16]. Gerra suggested the possibility that neglect by parents could partially contribute to complex neurobiological derangement, including HPA axis and dopamine system dysfunctions, playing a crucial role in addictive and affective disorder susceptibility. In summary, a variety of evidence has demonstrated that adolescents with parental absence and neglect showed symptoms of anxiety [69]. Moreover, we found a difference in anxiety levels among the 3 schools. This finding shows that the risk levels of individuals differ across school contexts/classrooms [70].

### Association between gaming disorder and insomnia

We found that GD led to a higher risk of insomnia. This finding is consistent with a previous study showing that excessive use of the internet plays a crucial role in initiating and increasing sleep problems among adolescents [71]. One explanation for this high correlation suggests indirect effects of smartphone addiction by poor self-regulation and bedtime procrastination on sleep quality [72]. A few physiological mechanisms might partly explain this finding. Previous research reported that school-aged children with GD have higher sympathetic activity and lower parasympathetic activity and overall autonomic activity [73]. Furthermore, acute sleep deprivation was associated with decreased parasympathetic activity [74]. In addition, some technical mechanisms could partly explain this result. Bright light exposure and radiation from electronic devices might suppress the release of melatonin and delay the circadian rhythm [75]. That is, blue light from smartphones might suppress sleep-promoting hormones (e.g., melatonin) [76]. In addition, exposure to bright light has been associated with the suppression of melatonin secretion and delayed sleep and wakefulness, which can increase consciousness and sleep disturbances [77].

We also found that those who were senior high school students had a more than one-fold increase in episodic insomnia compared to those who were junior high school students. One explanation of this phenomenon suggests that there is a dramatic change in sleep as a normal developmental process during adolescence. As adolescents age, their sleep–wake cycle tends to be delayed, sleep time decreases, and sleep architectural changes, such as slow wave sleep and slow wave activity, decrease [78]. While a large proportion of these sleep changes across adolescence are shown to be a consequence of normal brain development, more prevalent causes of sleep problems, especially short sleep duration, are caused by exogenous factors [79]. Therefore, effective early intervention for adolescents is essential for improving their mental health and resolving sleeplessness.

In addition, the present study specifically examined whether parental absence was a risk factor for insomnia in adolescents and found that exposure to parental absence leads to a higher risk of insomnia. These results are the same as those of previous studies [34]. The link between parental absence and insomnia can be explained by the absence of parents indicating poor supervision of children, and teens have poor self-control and are unable to follow a good sleep schedule. These individuals might be more likely to adopt behaviors that impair sleep, such as using their smartphones before sleep and spending excessive time on the internet [32, 80].

In summary, gaming disorder increases the risk of anxiety and insomnia. Due to different demographic measures, GD has different effects on anxiety and insomnia and presents increasing forms under different degrees of anxiety. The high incidence of GD among adolescents emphasizes the need for parents and schools to offer strong supervision and emotional support. For adolescents with GD, effective early intervention regarding smartphones should be valued. Furthermore, parents and schools should determine in a timely manner when teenagers have symptoms of anxiety or insomnia and provide timely and early emotional support and understanding.

## Limitations

Several limitations should be noted. First, we used a cross-sectional approach, which was unable to make causal inferences between GD and anxiety or insomnia. Second, we used a convenience sampling method to conduct this research in three high schools in Guangxi Province, China. Thus, the sample cannot represent all adolescents and might not be generalized to a conventional population. Third, since the assessments of GD, anxiety and insomnia were based only on the self-reported perspectives of teenagers, there is also a chance of recall bias on the part of the participants. In addition, the assessment tool for GD that we used was not sufficiently detailed to evaluate the severity, duration, or sequence of occurrence of the addiction. Fourth, some potentially important factors related to GD, anxiety, and insomnia were not collected adequately and thus could not be considered in the statistical analyses. For example, parenting styles and parental monitoring of smartphone use could affect adolescents’ GD behaviors. The present research was mainly exploratory to emphasize the relationships of GD with anxiety and insomnia. It is necessary to conduct further large sample research to obtain the causality of the relationships identified and the impacts of various variables.

## Conclusion

Our research reported that the incidence of GD is generally very high in the Chinese ethnic minority adolescent population. GD has a considerable effect on anxiety and insomnia. GD is a very strong risk factor for anxiety (whether it is mild, moderate or severe) and insomnia. Therefore, we recommend that game and smartphone use be carefully monitored in adolescents. It is essential to offer psychological support for adolescents who have GD since they are likely to have anxiety and insomnia. In addition, there remains a need for larger-scale research to determine not only causality but also other potential variables that might modify the relationship.

## Data Availability

The datasets analyzed during the current study are available from the corresponding author upon reasonable request.

## Abbreviations

GD: Gaming disorder
POGQ-SF: Problematic Online Gaming Questionnaire Short-Form
GAD-7: Generalized Anxiety Disorder Scale-7
AIS: Athens Insomnia Scale
ICD-11: International Classification of Diseases 11th edition
IGD: Internet gaming disorder
APA: American Psychiatric Association
DSM-5: Diagnostic and Statistical Manual of Mental Disorders Fifth Edition
